# HACE1 deficiency leads to structural and functional neurodevelopmental defects

**DOI:** 10.1212/NXG.0000000000000330

**Published:** 2019-04-29

**Authors:** Vanja Nagy, Ronja Hollstein, Tsung-Pin Pai, Michel K. Herde, Pisanu Buphamalai, Paul Moeseneder, Ewelina Lenartowicz, Anoop Kavirayani, Georg Christoph Korenke, Ivona Kozieradzki, Roberto Nitsch, Ana Cicvaric, Francisco J. Monje Quiroga, Matthew A. Deardorff, Emma C. Bedoukian, Yun Li, Gökhan Yigit, Jörg Menche, E. Ferda Perçin, Bernd Wollnik, Christian Henneberger, Frank J. Kaiser, Josef M. Penninger

**Affiliations:** From the IMBA (V.N., T.-P.P., P.M., A.K., I.K., R.N., J.M.P.), Institute of Molecular Biotechnology of the Austrian Academy of Sciences, VBC—Vienna BioCenter Campus, Austria; Department of Medical Genetics (J.M.P.), Life Science Institute, University of British Columbia, Vancouver, Canada; Ludwig Boltzmann Institute for Rare and Undiagnosed Diseases (V.N., E.L.), Vienna, Austria; Section for Functional Genetics at the Institute of Human Genetics (R.H., F.J.K.), University of Lübeck; German Center for Cardiovascular Research (DZHK e.V.) (F.J.K.), Partner Site Hamburg/Kiel/Lübeck, Lübeck; Institute of Cellular Neurosciences (M.K.H., C.H.), University of Bonn Medical School, Germany; Centre for Neuroendocrinology (M.K.H.), Department of Physiology, School of Biomedical Sciences, University of Otago, Dunedin, New Zealand; Department of Neurophysiology and Neuropharmacology (A.C., F.J.M.Q.), Center for Physiology and Pharmacology, Medical University of Vienna, Austria; Drug Safety and Metabolism (R.N.), IMED Biotech Unit, AstraZeneca, Gothenburg, Sweden; Division of Genetics and the Roberts Individualized Medical Genetics Center (M.A.D., E.C.B.), Children's Hospital of Philadelphia, PA; Departments of Pediatrics (M.A.D.), University of Pennsylvania Perelman School of Medicine, Philadelphia, PA; Institute of Human Genetics (Y.L., G.Y., B.W.), University Medical Center Göttingen, Germany; Institute of Neurology (C.H.), University College London, UK; German Center for Neurodegenerative Diseases (DZNE) (C.H.), Bonn, Germany; Zentrum für Kinder- und Jugendmedizin (G.C.K.), Neuropädiatrie, Klinikum Oldenburg, Germany; Department of Medical Genetics (E.F.P.), Faculty of Medicine, Gazi University, Ankara, Turkey; CeMM Research Center for Molecular Medicine of the Austrian Academy of Sciences (P.B., J.M.), Vienna, Austria.

## Abstract

**Objective:**

We aim to characterize the causality and molecular and functional underpinnings of *HACE1* deficiency in a mouse model of a recessive neurodevelopmental syndrome called spastic paraplegia and psychomotor retardation with or without seizures (SPPRS).

**Methods:**

By exome sequencing, we identified 2 novel homozygous truncating mutations in *HACE1* in 3 patients from 2 families, p.Q209* and p.R332*. Furthermore, we performed detailed molecular and phenotypic analyses of *Hace1* knock-out (KO) mice and SPPRS patient fibroblasts.

**Results:**

We show that *Hace1* KO mice display many clinical features of SPPRS including enlarged ventricles, hypoplastic corpus callosum, as well as locomotion and learning deficiencies. Mechanistically, loss of HACE1 results in altered levels and activity of the small guanosine triphosphate (GTP)ase, RAC1. In addition, HACE1 deficiency results in reduction in synaptic puncta number and long-term potentiation in the hippocampus. Similarly, in SPPRS patient–derived fibroblasts, carrying a disruptive *HACE1* mutation resembling loss of HACE1 in KO mice, we observed marked upregulation of the total and active, GTP-bound, form of RAC1, along with an induction of RAC1-regulated downstream pathways.

**Conclusions:**

Our results provide a first animal model to dissect this complex human disease syndrome, establishing the first causal proof that a HACE1 deficiency results in decreased synapse number and structural and behavioral neuropathologic features that resemble SPPRS patients.

Homologous to the E6-AP carboxyl terminus domain and ankyrin repeat containing E3 ubiquitin-protein ligase 1 (HACE1), was identified to be downregulated in a number of tumors, as well as to play a role in inflammatory responses.^1–4^ Analysis of *Hace1* knock-out (KO) mice showed HACE1 is ubiquitously expressed, with relatively high expression in the brain.^2^

A neurodevelopmental disorder named spastic paraplegia and psychomotor retardation with or without seizures (SPPRS) has recently been described in 15 patients of 6 unrelated families, associated with mutations throughout the *HACE1* gene.^5–7^ Mutations are predicted to be deleterious to HACE1 protein function, either disrupting folding or causing a frame shift and early termination of translation, resulting in no detectable protein product in patient-derived fibroblasts.^6,7^ Clinical manifestations are variable and include early-onset developmental delays, severe intellectual disability, epilepsy, hypotonia, spasticity, ataxia, and difficulty with verbal communication. MRI findings include enlarged ventricles, hypoplastic corpus callosum, and altered white and gray brain matter ratios. There are several known targets for HACE1 ubiquitination, including Ras-related C3 botulinum toxin substrate 1 (RAC1), small GTPase, important for different aspects of brain development and neuronal function.^4,8–11^ In addition to cytoskeletal remodeling, RAC1 can regulate reactive oxygen species (ROS) levels as part of the nicotinamide adenine dinucleotide phosphate oxidase (NADPH) complex.^12^ Correspondingly, *Hace1* KO mice were reported to have elevated ROS levels.^13^ While the role for *hace1* was recently described in embryonic development in *Xenopus laevis*^14^ and murine HACE1 has been implicated in Huntington disease,^13^ nothing is known about the role of HACE1 during mammalian nervous system development or the molecular underpinnings of SPPRS. To elucidate the neurodevelopmental pathophysiology of SPPRS, we therefore performed detailed analysis of *Hace1* KO mice and confirmed our findings in SPPRS patient–derived fibroblasts.

## Methods

See supplemental information (e-methods, links.lww.com/NXG/A151) for more details.

### Standard protocol approvals, registrations, and patient consents

Genetic and clinical data of patients 6–8 were published.^6^ Our newly identified patients are labeled as patients 9–11. Genetic data regarding patient 9 were produced as part of a clinical diagnostic service (GeneDx), and the research study including patients 10 and 11 has been approved by the ethics committee at The University of Göttingen. Biological materials of patients and healthy donors (HDs) and written informed consents were obtained in accordance with the Declaration of Helsinki. Fibroblasts from 2 female patients and 1 male patient only, 6, 7, and 10, respectively, are used in this study, as no consent was available for the others.

### Mice

Animals were housed at the Institute of Molecular Biotechnology, Vienna, Austria, maintained under a 12-hour light/dark cycle, and provided with food and water ad libitum. Experiments described in this study were approved by the Bundesministerium fur Wissenschaft, Forschung und Wirtschaft (BMWFW-66.015/0004-WF/V/3b/2015) and performed according to EU-directive 2010/63/EU.

### Immunoblotting

The following antibodies were used for standard Western blotting protocols: anti-HACE1 at 1:1,000 dilution (AbCam), anti-Cyclin D1 at 1:1,000 (AbCam), anti-Glyceraldehyde-3-Phosphate Dehydrogenase at 1:1,000 dilution (Cell signalling), anti-RAC1 at 1:1,000 dilution (Millipore), anti-ß-actin at 1:5,000 dilution (Sigma, clone AC-74), and appropriate secondary horseradish peroxidase–linked whole antibodies (GE Healthcare).

### Histology

Selected brains were isolated, processed, embedded, sectioned, and stained by the Histopathology Facility at the Vienna Biocenter Core Facilities (VBCF), member of the Vienna Biocenter (VBC), Austria. Briefly, 2-μm-thick coronal or sagittal paraffin-embedded sections were prepared by routine microtomy and stained with hematoxylin and eosin (H&E, Shandon* Harris Hematoxylin Acidified; Thermo Scientific Shandon Eosin Y; Fisher Scientific), Luxol Fast Blue–Cresyl Violet (LFBCV, Sigma), or with anti–Myelin Basic Protein antibody (Abcam, 1:100). Slides were imaged using a Zeiss Axioskop 2 MOT microscope (Carl Zeiss Microscopy) and subsequently digitized with the Pannoramic FLASH 250 II automated slide scanner (3D Histech). Images were acquired with the Pannoramic Viewer software (3D Histech) and with SPOT Insight camera (Diagnostic Instruments, Inc.).

### MRI

Anesthetized male C57Bl6/J mice 8–10 weeks of age and their *Hace1* KO littermates were imaged in the Preclinical Imaging Facility at VBCF (pcPHENO, VBCF), member of the VBC, Austria, using a 15.2 T MRI (Bruker BioSpec, Ettlingen, Germany). Values were normalized to brain size, averaged, and presented as percentage of total brain volume, and unpaired Student's *t* test was used to determine statistical significance.

### Behavioral testing

Open-field test (OFT) and elevated plus maze were performed as described previously using an automated activity system (TSE-Systems, Germany).^15^ Unpaired Student's *t* test was used to determine significance. Accelerating rotarod was performed on a 5–40 rpm accelerating apparatus (Ugo Basile), as described previously.^16^ Significance was determined by 2-way analysis of variance (ANOVA) with Sidak's multiple comparisons. In the ladder rung walking task (pcPHENO, VBCF), mice were trained to walk across a ladder (length: 80 cm; spacing of rungs: 1 cm) toward an escape ladder in the TSE MotoRater system (tse-systems.com). Mice were recorded and manually scored using SimiMotion software 8.5.0.327.^17^ Unpaired Student's *t* test was used to determine significance. T-maze was performed as described previously.^18^ Significance of deviation of the number of successes in mutant vs wild-type (WT) mice was evaluated using a binomial test. For acoustic fear conditioning (pcPHENO, VBCF), mice were trained to associate the conditioned sound stimulus (CS = 85 dB, 10 kHz) with the unconditioned foot shock stimulus delivered by the floor grid (US = 1.5 mA), as described previously, using Coulbourn Habitest operant cages (Coulbourn Instruments, MA and FreezeFrame, Actimetrics, IL).^19^ Unpaired Student's *t* test was used to determine significance. Mice were trained in the Morris water maze (pcPHENO, VBCF) as described previously.^20^ Unpaired Student's *t* test was used to analyze the short- and long-term memory data and 2-way ANOVA with Sidak's multiple comparison test for the latency to reach the platform. Mice were tested for startle responses and prepulse inhibition (PPI) as described previously^21^ using the SR-LAB Startle Response System (San Diego Instruments) chamber. Two-way ANOVA with Sidak's multiple comparison test was used to determine significance.

### Hippocampal slices preparation and electrophysiologic recordings

Electrophysiologic field recordings in the *stratum radiatum* of acute hippocampal slices were performed as described previously.^22^ Two-population Student's *t* test were used to determine significance.

### Synaptic number analysis

*Hace1* KO; Thy1-green fluorescent protein (GFP) line M and *hace1* WT; and Thy1-GFP line M^16^ were analyzed using the LSM700 Axio Imager. Synapse number counts and measurement of neurite lengths were performed using ImageJ.^23^ Statistical analysis was performed using Student's *t* test.

### Active RAC1 pulldown

Cellular levels of active RAC1 in fibroblasts were analyzed using the Active Rac1 Pull-Down and Detection Kit (Thermo Fisher Scientific) according to the manufacturer's instructions.

### Migration assay

Primary dermal fibroblasts from patients 6, 7, and 10 and HDs were seeded at high density in 24-well plates for 24 hours and scratched using a pipette tip. The area of the scratch at 0 and 24 hours following the scratch was measured digitally in pixels, and the relative gap closure was calculated and normalized to wild-type cells. Unpaired Student's *t* test was used to determine significance.

### ROS detection in the mouse brain and human primary dermal fibroblasts

Twenty-micrometer coronal mouse brain sections were washed 2X in phosphate buffered saline and incubated in 10-mM dihydroethidium for 15 minutes at 37°C. Sections were washed twice in phosphate buffered saline and imaged with a Zeiss Axioplan2 microscope. Levels of ROS in fibroblasts were investigated using CellROX Deep Red staining (Thermo Fischer) followed by flow cytometric analysis according to the manufacturer's instructions. Statistical analysis was determined using unpaired Student's *t* test.

## Results

### Identification of novel HACE1 mutations in SPPRS patients

Family 1 originating from Saudi Arabia had 1 affected female individual, patient 9, born to healthy, consanguineous parents (figure e-1A, links.lww.com/NXG/A148). The patient presented with similar clinical symptoms as previously reported cases of SPPRS, including severe intellectual disability, developmental delay, and inability to sit or speak by the age of 5 years.^6^ Cranial MRI revealed microcephaly and brachycephaly, as well as hypoplastic corpus callosum and likely brainstem abnormality, small sella with ectopic neurohypophysis, and mild ventriculomegaly (figure e-1B). Additional clinical findings include mild facial dysmorphia, skeletal abnormalities, and ulnar deviation of the wrists and small feet (figure e-1C). In an unrelated consanguineous family 2 originating from Turkey, born to a healthy father and mother with Hashimoto disease are one female (patient 10) and one male (patient 11), 4 and 6 months of age at initial admission (Figure e-1D). During pregnancy, the mother was euthyroid and both children were carried to term. Patients had variable clinical symptoms in comparison with patient 9 and those previously reported, including hypotonia, small feet, enlarged head circumference, inverted and wide spaced nipples, facial dysmorphic features, and strabismus (figures e-1, E and F). Considering the pronounced hypotonia in all 3 patients, ataxia was difficult to determine. For detailed clinical symptoms of all patients, please refer to table.

**Table:**
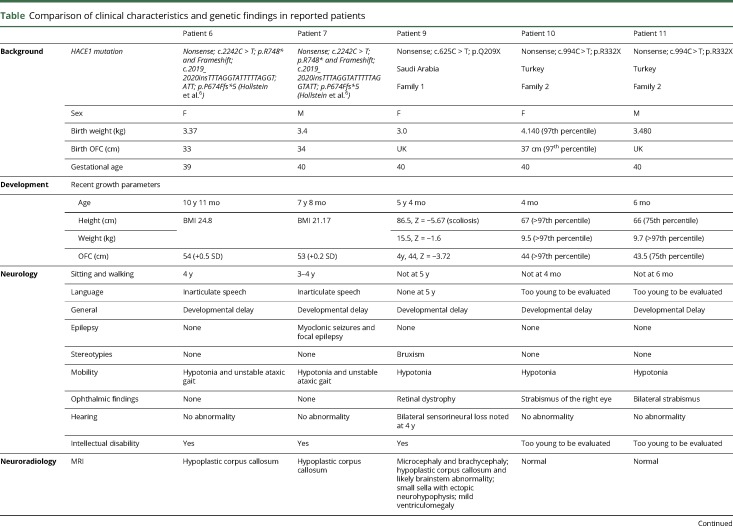

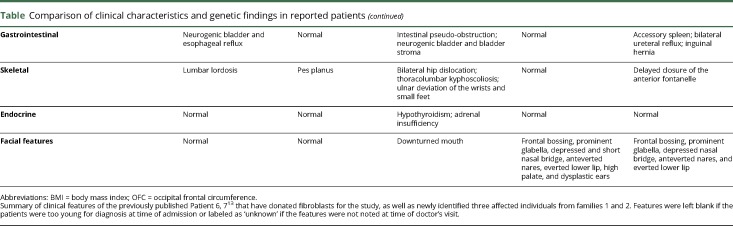
Comparison of clinical characteristics and genetic findings in reported patients

**Figure 1:**
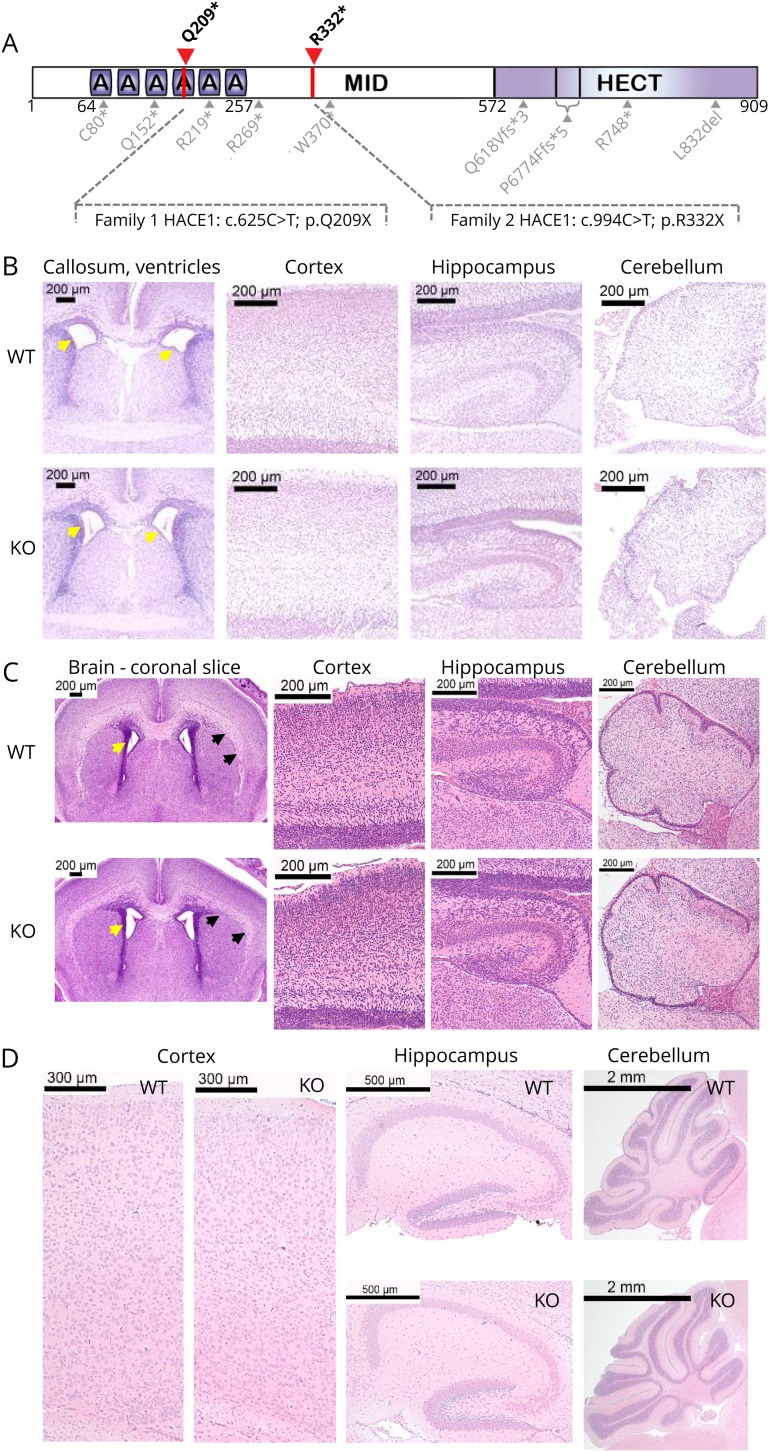
Histologic analysis of brain regions in neonatal and young *Hace1* knock-out (KO) mice (A) Schematic representation of HACE1 protein and locations of mutations of previously published (in gray) and novel mutations reported here (red arrows, black letters). (B) H&E-stained sections of wild-type (WT) and *Hace1* KO mice at E18.5 reveal no major structural abnormality across the regions studied, with slight ventriculomegaly in the *Hace1* KO brain as compared with controls, left panel (yellow arrows). (C) H&E-stained coronal sections of the newborn brain of WT and *Hace1* KO mice at P0.5 reveal ventricular dilation in the *Hace1* KO brain (yellow arrows) and attenuation of the corpus callosum (black arrows). (D) H&E-stained sections of WT and *Hace1* KO mice at P14.5. Cortical lamination, hippocampal formation, and the cerebellar vermis are comparable between WT and *Hace1* KO in all 3 age groups studied. All brain regions are labeled in the figure and magnifications are as indicated. A = ankyrin domain; H&E = hematoxylin and eosin; HECT = homologous to the E6-AP carboxyl terminus; MID = middle region.

In these 3 patients, mutations in *HACE1* were identified by exome sequencing and verified by Sanger sequencing: patient 9 carries a homozygous c.625C>T; p.Q209* *HACE1* mutation in exon 8, NM_020771.3:c.625C>T, p.Q209* (chr6:104 797 018 on the hg38 build); patients 10 and 11 carry a homozygous c.994C>T; p.R332* *HACE1* mutation in exon 11, NM_020771.3:c.994C>T, p.R332* (chr6:104 791 584) (figure 1A). Both are nonsense mutations predicted to result in a truncated HACE1 protein product with no catalytic activity. Parents in both families were determined to be heterozygous carriers. Figure 1A also indicates all previously reported *HACE1* mutations in SPPRS patients.^5,6^ Thus, we have identified three new cases with biallelic HACE1 mutations associated with marked and diverse neurologic as well as non-neurologic abnormalities, diagnosed to be SPPRS.

### *Hace1*-mutant mice exhibit brain morphologies similar to SPPRS patients

To determine whether *Hace1* KO mice develop any clinical symptoms of SPPRS patients, we first analyzed structural and cellular features of mutant mouse brains using 15.2 T MRI and/or histologic staining at different developmental stages. Histologic examination of mouse brains at embryonic day (E) 18.5, postnatal day (P) 0.5, and P14.5 revealed no evident defects cortical lamination, cerebellar foliation, hippocampal architecture, or cellular aberration and density in any developmental period examined (figure 1, B–D). However, enlarged ventricles and hypoplastic corpus callosum (figure 1, B and C) were noted in *Hace1* KO animals as compared with WT littermates, as early as E18.5 and P0.5, respectively.

**Figure 2:**
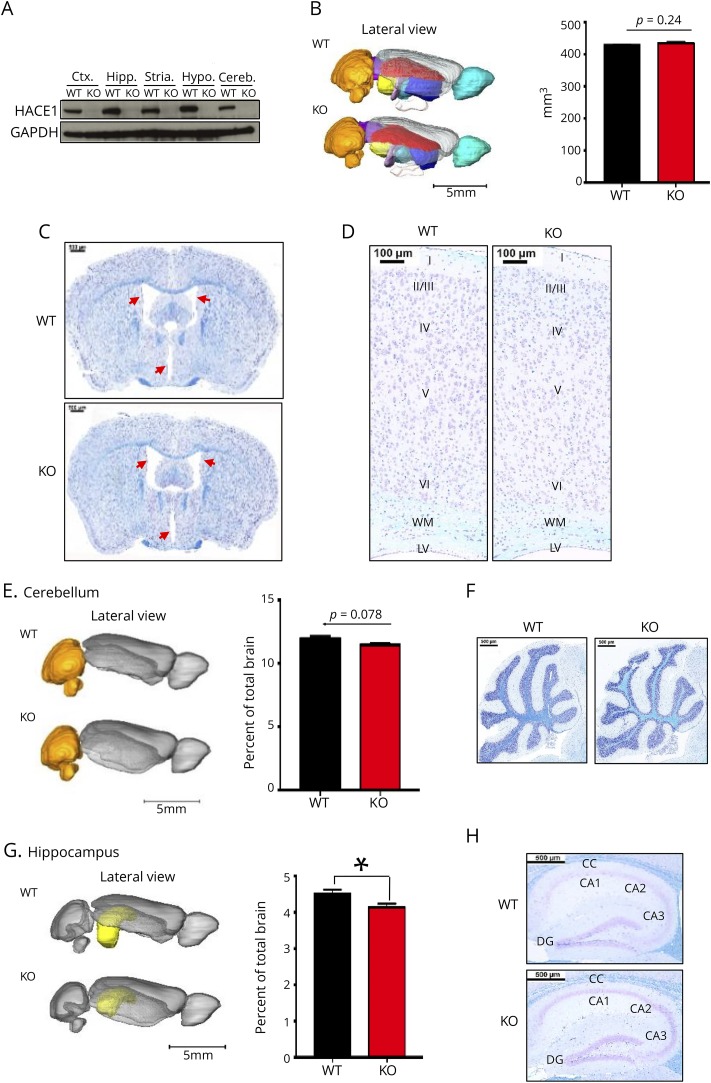
Morphology of the *Hace1* KO mouse brain (A) Western blot analysis depicting HACE1 abundance in the indicated 8–12-week-old male mouse brain regions of wild-type (WT) and *Hace1* knock-out (KO) mice. (B) Pseudocolored 3D reconstruction of lateral MRI images of representative whole mouse brains, and quantification (mean values ± SEM) showing no difference in total brain volumes in 8–12-week-old male WT and *Hace1* KO littermates. Orange—cerebellum, yellow—hippocampus, red—corpus callosum, light blue—thalamus, purple—colliculi, turquoise—olfactory bulbs, and dark blue—putamen; WT n = 12 and KO n = 9; *p* value as indicated in the bar graph; unpaired Student's *t* test. (C) LFB-CV–stained coronal sections of whole WT and *Hace1* KO adult mouse brains. Ventriculomegaly (red arrows) is evident in the KO brain as compared with the WT littermate. (D) LFB-CV–stained coronal sections of the adult cortex show no defects in cellularity or lamination (laminae I to VI) in *Hace1* KO mice. (E) Pseudocolored (orange) 3D reconstructed MRI images of representative lateral views of WT and *Hace1* KO littermate cerebelli showing no significant volumetric differences in *Hace1* KO vs WT control littermates. Right, quantification of volumetric differences; mean values ± SEM; WT n = 12; KO n = 9; *p* value as indicated in the figure; unpaired Student's *t* test. (F) Selected LFB-CV–stained sagittal sections of the cerebellum showing no apparent differences between WT and *Hace1* KO brains. (G) Pseudocolored (yellow) 3D reconstruction of MRI images of representative lateral view of hippocampi of WT and *Hace1* KO mice shows a significant reduction in hippocampal volume in *Hace1* KO brains; quantification is shown in the right panel; mean values ± SEM; WT n = 12 and KO n = 9; *p ≤ 0.05; unpaired Student's *t* test. (H) LFB-CV–stained coronal sections of the hippocampus show no notable morphological difference in cellularity between WT and *Hace1* KO brains. Scale bars as labeled in the images. CA = cornu ammonis; CC = corpus callosum; cereb = cerebellum; Ctx = cortex; DG = dentate gyrus; hipp = hippocampus; hypo = hypothalamus; LFB-CV = luxol fast blue - cresyl violet; LV = lateral ventricle; stria = striatum; WM = white matter.

Western blot analyses of 8-week-old mouse brain lysates revealed HACE1 to be expressed throughout the adult mouse brain, which was absent in the brains of *Hace1* KO mice (figure 2A). Total volume and morphology of the *Hace1* KO adult mouse brain as compared with WT littermates measured by MRI and/or histologic examination revealed no whole-brain volumetric or cellularity differences as compared with WT littermates (figure 2, B and C). Review of histologic sections also revealed no abnormality in cortical laminar organization and cellular density in the adult brain (figure 2D). Detailed analysis of different brain regions via MRI also showed no significant differences in the volumes of the cortex, colliculi, thalamus, and putamen, comparing adult WT and *Hace1* KO brains, with a small decrease in the volume of the olfactory bulb (data not shown). The adult cerebellar volume and foliation as well as cortical laminar organization were comparable between *Hace1* KOs and their control WT littermates (figure 2, E and F). Hippocampal volume was significantly decreased as measured by MRI (figure 2G); however, there were no evident aberrations in cellularity and architecture on microscopic examination of hippocampi in adult animals (figure 2H).

Of interest, volumetric MRI reconstructions revealed a significant reduction in whole-brain white matter (figure e-2, A–D, links.lww.com/NXG/A149) in *Hace1* KO adult mice as compared with their WT littermates, substantiated by myelin basic protein staining and volumetric MRI analysis (figure e-2, B–D), similar to findings in reported SPPRS patients^5–7^ and patient 9 described here (figure 1C and table). Also, noted was a reduction of the anterior commissure in *Hace1* KO mice as compared with WT littermates (figure e-2C). In addition, in line with findings in SPPRS patients, as well as adult and developing mouse brains, we observed enlarged ventricles in *Hace1* KO mice as compared with control littermates (figure 2C). These data show that *Hace1* KO mice phenocopy key structural features associated with SPPRS patients.

### HACE1*-*deficient mice exhibit locomotion and associative learning disabilities

**Figure 3:**
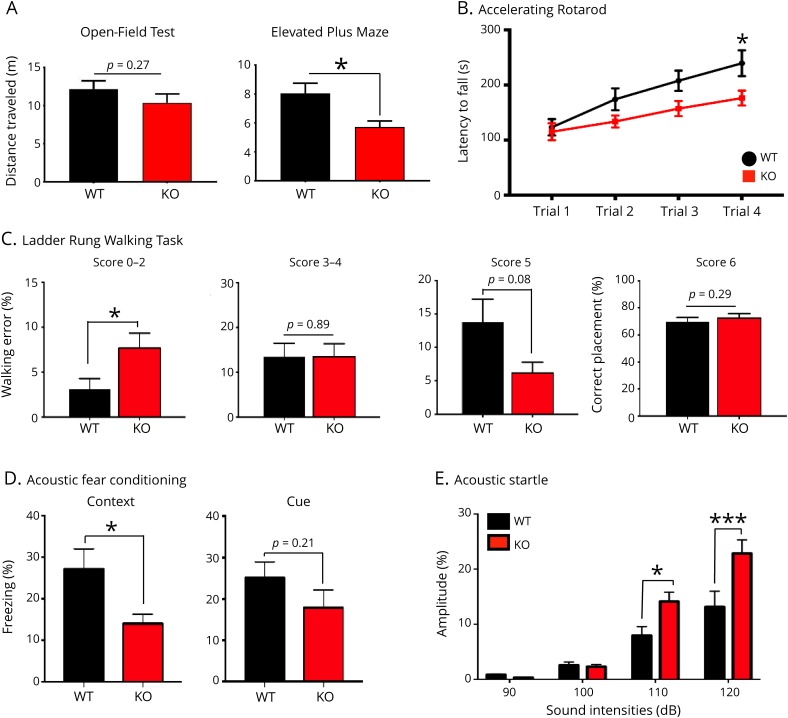
Behavioral analysis of *Hace1* KO mice reveals deficits in specific locomotion, learning, and memory tasks (A) Open-field maze (OFM) and elevated plus maze (EPM) showed slight and significant reductions in the distances *Hace1* KO mice had traveled as compared with their WT littermates, respectively; data are shown as mean values ± SEM; WT n = 10 and KO n = 10; *p ≤ 0.05 or as indicated in the bar graphs; unpaired Student's *t* test. (B) *Hace1* KO mice show significantly shorter latencies to fall off the accelerating rotarod; mean values ± SEM; WT n = 19 and KO n = 21; *p ≤ 0.05; 2-way ANOVA with Sidak's multiple comparison test. (C) Ladder rung walking task illustrates a significant increase in the percentage of total misses, deep slips, and/or slight slips (0–2 score) in *Hace1* KO mice compared with WT littermate controls; mean values ± SEM; WT n = 12 and KO n = 9; *p ≤ 0.05; unpaired Student's *t* test. No significant differences were noted in the 3–4 score (replacement and correction), 5 score (partial placement), or 6 score (correct placement). Bar graphs depict mean values of all paws ± SEM; *p* values are indicated in figures; WT n = 12 and KO n = 9; unpaired Student's *t* test. (D) Significant deficiency in long-term contextual memory as measured by auditory fear conditioning was detected in *Hace1* KO mice as compared with WT littermates (left panel); mean values ± SEM; WT n = 16 and KO n = 13; *p ≤ 0.05; unpaired Student's *t* test. Cued fear conditioning was not significantly different in *Hace1* KO mice as compared with WT littermates; mean values ± SEM; WT n = 16; KO n = 13; *p* value = 0.21; unpaired Student's *t* test. (E) *Hace1* KOs show significant reduction in acoustic startle inhibition at 110 and 120 dB as compared with WT littermates. Mean values ± SEM; WT n = 14; KO n = 11; *p ≤ 0.05; ***p ≤ 0.001; 2-way ANOVA with Sidak's multiple comparison test. ANOVA = analysis of variance; KO = knock out; WT = wild type.

To assess what, if any, behavioral defects can be detected in *Hace1* KO mice, we subjected cohorts to an extensive battery of behavioral assays. The most notable clinical features of SPPRS are intellectual disability and ataxia^5–7^; we therefore focused on hippocampal- and cerebellar-dependent tasks, principal brain regions regulating those behaviors in mice. *Hace1* KO mice generated in our group previously are generally healthy, viable, and fertile.^2^ First, we found no significant differences in anxiety levels in *Hace1* WT and KO littermates analyzed by the OFT and the elevated plus maze (figure e-3, A and B, links.lww.com/NXG/A150); however, we noted a reduction in the distance traveled during the testing (figure 3A). These data indicated that while our mice exhibited normal anxiety levels, there may be a locomotion defect. Therefore, we tested *Hace1* KO mice for any locomotion and gait defects. WT and *Hace1* KO mice were tested in the accelerating rotarod task, a standard assay to determine locomotion and gait defects.^16^ While *Hace1* KO mice and control littermates had similar latencies to fall off the rotating rod in the first trial, *Hace1* KO mice exhibited shorter latencies to fall on all subsequent trials (figure 3B). We further analyzed the mice on a ladder rung walking task, which enables the observer to analyze coordinated front and hind paw placement on each individual rung of the ladder.^17^ While *Hace1* KO animals were capable of performing the task without falling off the apparatus and showed similar percentages of correct placement over all limbs analyzed, percent of major missteps made were significantly higher as compared with WT littermates. Both groups of animals had similar placement and correction scores, while WT animals had a higher percentage of partial placement on rungs (figure 3C). Thus, *Hace1* KO mice exhibit locomotion and gait deficits.

The second notable clinical feature of SPPRS patients is intellectual disability (table).^5–7^ To determine whether learning and memory processes are altered in *Hace1* KO animals, we analyzed mutant mice in various hippocampal-dependent behavioral tasks. In the Morris water maze, KO mice were able to swim normally and there were no apparent differences in latencies between WT and *Hace1* KO littermates to find the visible platform (not shown), in escape latencies during any of the training days (figure e-3C, links.lww.com/NXG/A150), nor in short- or long-term memory (figure e-3C, right panels). Likewise, there were no differences in T-maze between WT and *Hace1* KO animals (figure e-3D). However, subjecting the animals to cued and contextual fear conditioning revealed significantly reduced freezing responses in the contextual aspect of the task in *Hace1* KO mice compared with WT littermates (figure 3D).

In addition, *Hace1* KO mice exhibited significantly enhanced startle amplitude responses or reduced startle inhibition, in the acoustic startle task at presentation of 110- and 120-dB startle sounds as compared with their WT littermates (figure 3E). These results also suggest *Hace1* KO animals do not have any hearing deficiencies. PPI was reduced for all prepulse intensities presented (80, 85, 90, and 95 dB); however, this reduction was not significant when comparing *Hace1* KO animals with WT controls (figure e-3E, links.lww.com/NXG/A150). These data indicate that, besides locomotion defects, associative learning is altered in *Hace1* KO mice, paralleling behavioral findings in human SPPRS patients.

### *Hace1* deficiency results in altered hippocampal synaptic transmission and reduced synapse numbers

**Figure 4:**
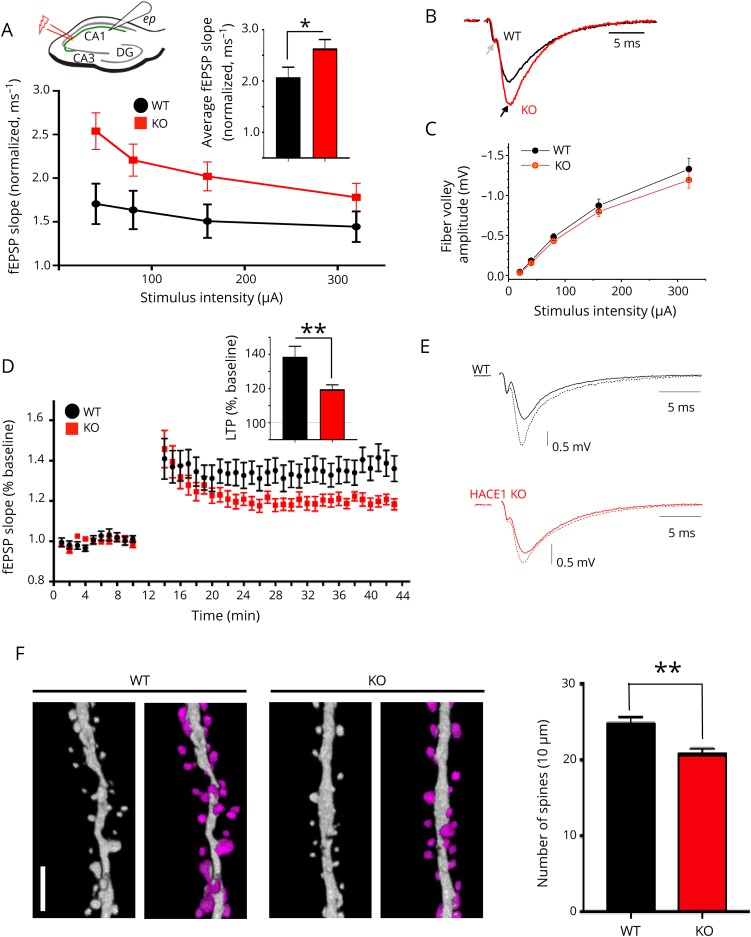
Altered long-term potentiation and reduced hippocampal synapse numbers in *Hace1* KO mice as compared with WT controls (A) Upper left panel, schematic fEPSP recording configuration from acute hippocampal slices: stimulation (red bolt) of CA3-CA1 Schaffer collaterals (green), the fEPSP extracellular recording electrode in the CA1 region (white cone, ep = extracellular pipette). Synaptic transmission at CA3-CA1 hippocampal synapses is increased in *Hace1* KO mice compared with WT littermates. fEPSP slopes were normalized to axonal fiber volleys to account for variations in axonal stimulation efficacy between slice preparations and animals. Analysis was performed over a range of stimulus intensities. Average fEPSP slopes were significantly higher in *Hace1* KO slices; mean values ± SEM; WT n = 19 and KO n = 20; *p ≤ 0.05; 2-population Student's *t* test. (B) Representative traces of fEPSPs in response to CA3-CA1 synapse stimulation obtained from WT (black trace) and *Hace1* KO (red trace) mice. Field EPSP (black arrow) recordings were normalized to fiber volleys (gray arrow) to account for varying stimulation efficiencies across experiments and preparations. Time scale of recording is indicated. (C) No significant difference was found between fiber volley amplitudes (mean values ± SEM) between the indicated genotypes; WT n = 19 and KO n = 20. (D) Long-term potentiation (LTP) of synaptic transmission was induced by HFS after a 10-minute baseline recording. fEPSP slopes were normalized to their baseline levels and plotted over time. LTP was reduced in *Hace1* KO animals; mean values ± SEM; WT n = 19 and KO n = 18; **p ≤ 0.01; 2-population Student's *t* test. (E) Representative fEPSP traces illustrating LTP experiments displayed in panel (D). Solid lines represent baseline fEPSPs, and dashed lines represent fEPSPs 30 minutes after LTP induction (*Hace1* WT: black, upper panel; *Hace1* KO: red, lower panel). (F) Representative 3D composite of z-stacked ×63 magnified (zoom factor of 2) confocal images of hippocampal CA1 pyramidal cell distal dendrites of *Hace1*^+/+^;*Thy1-GFP* and *Hace1*^*−/−*^;*Thy1-GFP* mice. Uncolored dendritic segments are on the left, synaptic spines are pseudocolored in magenta on the right panels; scale bar is 2.5 μm. Right bar graphs show quantifications of spine numbers; mean values ± SEM; WT n = 3 and KO n = 3; **p ≤ 0.01; unpaired Student's *t* test. fEPSPs = field excitatory post synaptic potentials; KO = knock out; WT = wild type.

We and others have previously shown that altered synaptic transmission and plasticity at CA3-CA1 Schaffer collaterals can underlie deficits of contextual fear memory.^22,24^ We tested whether synaptic transmission and plasticity of this pathway are affected in *Hace1* KO mice. Recordings of field excitatory post synaptic potentials in response to CA3-CA1 synapse stimulation (schematic of the set-up in figure 4A) revealed an upregulation of excitatory synaptic transmission in hippocampal slices from *Hace1* KO mice (figure 4, A and B). Further analysis showed that the increase in synaptic transmission at the CA3-CA1 synaptic pathway was most likely due to a postsynaptic effect of the *Hace1* deletion because neither the axonal fiber volley (figure 4C) nor the paired-pulse ratio, an indirect measure of the presynaptic release probability,^25^ was affected (interstimulus interval 50 ms, paired-pulse ratio: WT 1.70 ± 0.066, n = 19, *Hace1* KO 1.66 ± 0.036, n = 20, *p* = 0.64, 2-population *t* test, not shown). We then probed long-term potentiation (LTP), a cellular correlate of memory formation,^26^ of this synaptic pathway and found that LTP is significantly reduced in acute hippocampal slices from *Hace1* KO mice compared with WT littermates (figure 4, D and E). Of note, nerve conduction velocity was not affected in *Hace1* KO recordings as compared with controls (data not shown), in line with the report that nerve conduction velocity is unaffected in SPPRS patients.^6^

Because we observed hippocampal-dependent contextual fear learning deficiency and dysfunctions in electrophysiologic properties of pyramidal cells within the CA1 region of the hippocampus, we analyzed morphological properties of pyramidal neurons in this brain region. To this end, we crossed *Hace1* KO mice with Thy1-GFP-M mice to drive neuronal GFP expression, which allowed us to visualize neuronal morphology, including spines along dendrites of CA1 pyramidal cells.^27^ Quantification of selected dendritic segments indeed revealed a significant reduction in spine numbers in *Hace1* KO neurons as compared with hippocampal neurons from WT littermates (figure 4F). Whether such reduction in the synapse number is also present throughout the brain and might explain defective locomotion in *Hace1* KO mice needs to be determined. These data indicate that, as a correlate to hippocampus-dependent impaired associate learning, HACE1 deficiency leads to a deficit of synaptic plasticity and a marked reduction of synapses in the hippocampus.

### Elevated levels of active Rac1 in *Hace1* KO mouse brains and SPPRS patient–derived fibroblasts

**Figure 5:**
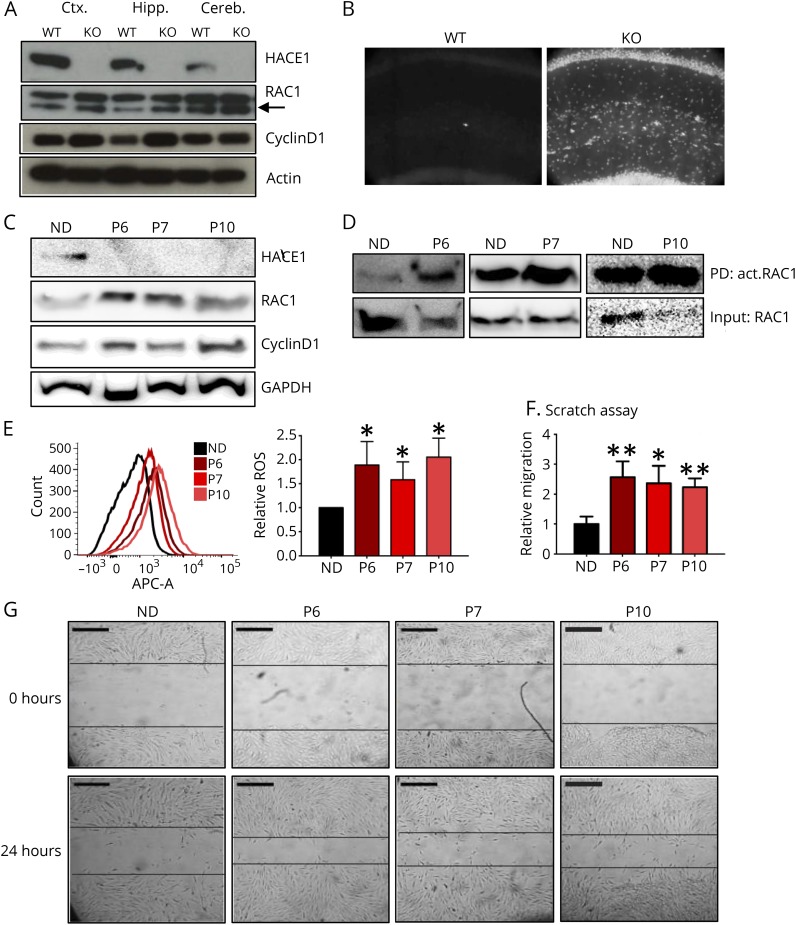
*Hace1* KO mouse brains and SPPRS patient–derived fibroblasts have elevated Rac1 and ROS levels (A) Western blot analysis of the cortex (ctx.), hippocampus (hipp.) and cerebellum (cereb.) from WT and *Hace1* KO mice revealed a strong upregulation of total RAC1 abundance (lower band, arrow) and Cyclin D1 protein in the absence of HACE1, in all indicated brain regions tested. Actin was used as a loading control. (B) ROS (detected by DHE staining) is elevated in the hippocampus of *Hace1* KO mice as compared with WT littermates; ×20 magnification. (C) Fibroblasts harvested from SPPRS patients (P6, P7, and P10) have no detectable HACE1 protein product as compared with normal donor fibroblasts (ND) as revealed by Western blotting using an antibody recognizing the HACE1 C-terminus; however, patient fibroblasts express elevated total abundance of RAC1 and Cyclin D1 protein product. GAPDH was used as a loading control. (D) SPPRS patient–derived fibroblasts exhibit elevated abundance of active RAC1 in comparison with normal donor fibroblasts, as measured by the *Active RAC1 Pull-Down and Detection Kit*. (E) Left panel, FACS analysis of SPPRS patient–derived fibroblasts incubated with CellROX deep red reagent revealed significantly more ROS in patient cells as compared with normal donor (ND) fibroblasts. Right panel, quantification of ROS production in the indicated patient and control fibroblasts; mean values ± SD; n = 7 for ND, P6, and P7 fibroblasts, and n = 3 biological replicates for patient 10 fibroblasts; *p ≤ 0.05; unpaired Student's *t* test. (F and G) Confluent SPPRS patient P6, P7, P10 and ND-derived fibroblasts were subjected to scratch assays, and migration was monitored. Panel (F) shows a quantification of fibroblast migration rates; mean values ± SD; n = 3; **p ≤ 0.01 and ***p ≤ 0.001 comparing patient fibroblasts with the ND controls; unpaired Student's *t* test. Representative images are shown in (G). KO = knock out; ROS = reactive oxygen species; SPPRS = spastic paraplegia and psychomotor retardation with or without seizures; WT = wild type.

To determine molecular alterations downstream of HACE1 deficiency, we focused on Ras-related C3 botulinum toxin substrate 1, RAC1, a well-characterized substrate for HACE1-mediated proteasomal degradation.^8,9^ Indeed, Western blot analysis showed that RAC1 protein abundance was increased in *Hace1* KO brains when compared with WT littermates (figure 5A). In addition, Cyclin D1 abundance and ROS levels, as downstream read-outs for RAC1 activity,^12^ were likewise increased in *Hace1* KO brain regions as compared with controls (figure 5, A and B).

The role of RAC1 has been extensively studied in the context of mouse brain development.^10,11^ Of interest, perturbation of RAC1 levels, either overexpression or downregulation, results in decrease in dendritic spines in the mouse brain, similar to our *Hace1* KO mice.^10,28^ To determine whether RAC1 is also changed in *HACE1*-mutant SPPRS patients, we performed Western blotting analysis of SPPRS patient–derived fibroblasts, isolated from patients 6, 7, and 10 (table). Of note, in these patients, the mutations result in undetectable HACE1 protein expression (figure 5C), resembling our *Hace1* KO mice. In all patients' fibroblasts, total abundance of RAC1 and Cyclin D1 was increased as compared with fibroblasts from HD controls (figure 5C). It is important that increased abundance of active GTP-bound RAC1 was detected in all 3 patient fibroblasts (figure 5D). As a functional consequence of increased RAC1 activity, ROS upregulation was also evident in SPPRS patient–derived fibroblasts (figure 5E). In addition, in a scratch assay, patient fibroblasts migrated significantly faster, as compared with control fibroblasts (figure 5, F and G), confirming enhanced RAC1 activity.^29–32^ These data show that loss of HACE1 expression results in increased levels of active RAC1 in both *Hace1* KO mouse brains and *HACE1*-mutant SPPRS patient–derived fibroblasts.

## Discussion

Here, we report 2 novel mutations in *HACE1*, p.Q209* and p.R332*, discovered in 3 patients from 2 unrelated consanguineous families with a complex neurodevelopmental disorder. These new patients have variable clinical symptoms, overlapping with previous descriptions of SPPRS.^5–7^ The genotype–phenotype correlations of the different mutations need to be addressed in future studies, as more SPPRS patients are diagnosed. We find that HACE1 is expressed throughout the adult mouse brain. *Hace1* KO mice have defects in basic sensorimotor processing, deficiencies in specific learning and memory tasks, reduced LTP of hippocampal CA3-CA1 synapses, and fewer synaptic spines at CA1 pyramidal neurons. In addition, they exhibit locomotion defects as compared with littermate controls. Furthermore, we show a marked upregulation of RAC1 levels throughout the mutant mouse brain and elevated ROS levels. Verifying that our findings are relevant in humans, active RAC1 abundance, downstream signaling components, ROS production, and cellular migration in SPPRS patient–derived fibroblasts are likewise dysregulated, all indicative of a hyperactive RAC1 pathway. Thus, HACE1 deficiency leads to neuroradiologic and behavioral manifestations in mice reminiscent of the clinical features seen in SPPRS patients.

A well-characterized target for HACE1 ubiquitination and subsequent proteasomal degradation is RAC1, a small member of the Rho family of GTPases, critical for neurogenesis, migration, axonal elongation, synaptogenesis, and activity-driven plasticity.^11^ HACE1 is therefore poised to regulate many functions of RAC1, by modulating its active GTP-bound levels, including NADPH-dependent ROS production.^12,13^

Mutations in several genes encoding guanine exchange factors and GTPase-activating proteins that regulate the on/off state of RAC1 have been identified to cause rare neuronal disorders.^33,34^ Indeed, analysis of a protein–protein interaction subnetwork extracted from the human-integrated protein–protein interaction reference database^35^ reveals RAC1 to have 25 first-order interactors reported to be causative of intellectual disabilities. Neuronally expressed RAC1 first-order interactors are enriched in GTPase signal transduction by Gene Ontology analysis. Furthermore, RAC1 was also identified as a candidate gene for intellectual disability in a meta-analysis of several thousand trios,^36^ and RAC1 missense mutations were recently reported to cause a neurodevelopmental disorder with variable symptoms in 7 individuals, some of which are overlapping with SPPRS patients.^37^ Of interest, mutations in RAC1 caused remarkably different neurologic phenotypes that ranged from microcephaly to normal head circumference to macrocephaly, as well as variable intellectual disability (4/7), hypotonia (4/7), epilepsy (3/7), behavioral problems (3/7), and stereotypic movements (2/7).^37^ The individuals who were determined to carry gain-of-function RAC1 mutations (3/7) had normal head circumference (1/3) or megalocephaly (2/3), in contrast to the 3 newly reported SPPRS patients here, who either had microcephaly (1/3) or normal head circumference (2/3). Hypoplastic corpus callosum, hypotonia, intellectual disability, and hearing impairment were noted in some but not all patients carrying gain-of-function RAC1 mutations, requiring further examination of the specific or common molecular pathology of RAC1-associated neurodevelopmental disorder and SPPRS.

Importantly, it has been previously established that dysregulation of RAC1 abundance in mouse models results in a decrease in dendritic spine number as well as reduction in hippocampal plasticity, as we have uncovered in our *Hace1* KO mice.^10,28,38^ We therefore speculate that RAC1 is a key factor underlining neuronal pathology in *Hace1* KO mice and HACE1-deficient patients. Whether RAC1-dependent cytoskeletal modulation and/or ROS homeostasis is the key determinant feature of HACE1-dependent neurodevelopmental deficiencies remains to be elucidated. The present study adds HACE1 to the large RAC1 regulatory complex, which when perturbed has deleterious consequence to brain development and neuronal function.

## Glossary

ANOVA: analysis of variance
HACE1: HECT Domain And Ankyrin Repeat Containing E3 Ubiquitin Protein Ligase 1
HD: healthy donor
KO: knock out
LTP: long-term potentiation
NADPH: nicotinamide adenine dinucleotide phosphate oxidase
OFT: open-field test
PPI: pre-pulse inhibition
RAC1: Ras-related C3 botulinum toxin substrate 1
ROS: reactive oxygen species
SPPRS: spastic paraplegia and psychomotor retardation with or without seizures
WT: wild type
